# Marching across and beyond West Africa: First record of the stem-galling fly *Cecidochares connexa* (Diptera: Tephritidae) in Central Africa and the implications for biological control of *Chromolaena odorata* (Asteraceae)

**DOI:** 10.1371/journal.pone.0252770

**Published:** 2021-06-04

**Authors:** Pascal O. Aigbedion-Atalor, Itohan Idemudia, Medetissi Adom, Ethelyn E. Forchibe, Hospice Tossou, David D. Wilson, Michael D. Day

**Affiliations:** 1 Centre for Biological Control, Department of Zoology and Entomology, Rhodes University, Makhanda (Grahamstown), South Africa; 2 African Regional Postgraduate Programme in Insect Science, University of Ghana, Legon, Ghana; 3 Department of Agriculture and Fisheries, Brisbane, Qld, Australia; USDA Forest Service, UNITED STATES

## Abstract

The Neotropical invasive plant *Chromolaena odorata* R.M. King and H. Robinson (Asteraceae) is a serious weed in West and Central Africa and two biological control agents that have been introduced into West Africa to help reduce its impacts on agriculture and biodiversity, have established. The stem-galling fly, *Cecidochares connexa* (Macquart) (Diptera: Tephritidae), has spread widely across West Africa since its release in only Côte d’Ivoire, occurring in six countries. This study aimed to investigate whether the gall fly had spread further across West Africa and into Central Africa. Here, we surveyed *C*. *odorata* for *C*. *connexa* galls in Cameroon between October 2018 and October 2020, along roadsides, on farms, residential areas, and abandoned plots, encompassing various vegetation types. Additional surveys were conducted across four countries (Ghana, Togo, Benin Republic and Nigeria) in West Africa that we considered the probable pathway for the spread of the gall fly into Central Africa. *Cecidochares connexa* was present at five of the six locations surveyed in Cameroon, albeit in varying abundance. In Africa, these findings represent the first-ever report of *C*. *connexa* outside of West Africa. In West Africa, we recorded significant expansion in the geographic range of *C*. *connexa*, as reflected in the absent-present record of *C*. *connexa* in two locations in Nigeria and one in Ghana, as well as its occurrence in all locations surveyed in Benin Republic and Togo. Clearly, Ghana, Togo, Benin Republic and Nigeria served as the dispersal pathway of *C*. *connexa* from the release sites in Côte d’Ivoire into Cameroon, covering over 2,300 km. Following the spread and establishment of *C*. *connexa* into Cameroon, we anticipate that it will continue to spread further into other parts of Central Africa which are climatically suitable. *Cecidochares connexa* is currently the only biological control agent for *C*. *odorata* in Central Africa. Given that it has significantly reduced populations of *C*. *odorata* in other countries where it has established, it is expected to have a similar impact in Central Africa.

## Introduction

Novel environments have increasingly become susceptible to biological invasions due to human-mediated activities, encompassing movement, global trade, and climate change, and there is no apparent indication of this abating [1–4]. These scenarios are exemplified by the perennial scrambling invasive alien plant *Chromolaena odorata* R.M. King and H. Robinson (Asteraceae) in West and Central Africa [5, 6]. The founder population of *C*. *odorata*, which is believed to have been introduced accidentally into Nigeria through an imported consignment from Sri Lanka in the late 1930s [7], successfully established and quickly became pervasive across West and Central Africa, with devastating impacts on biodiversity and agriculture [5]. *Chromolaena odorata* is still spreading and is now present in 12 of the 16 countries in West Africa, following the recent report of its occurrence in Guinea-Bissau [8], and it has been reported in seven of the eight countries in Central Africa [6, 9].

Physical control methods, encompassing slashing, uprooting, and burning the plant, were widely adopted for the control of *C*. *odorata* in the early phase of its invasion [10, 11]. Although effective, it became clear that these control methods were too laborious and not feasible to manage the unrelenting spread and increasing density of the weed in West and Central Africa [10, 11]. Consequently, in the 1970s, the Nigerian Government initiated a project to search for promising classical biological control candidates of *C*. *odorata* in its native range [10]. The initial project was unsuccessful but a renewed programme in the early 1990s, coordinated by the University of Guam (USA) and the Crop Research Institute (CRI) in Ghana, resulted in the release and establishment of the moth *Pareuchaetes pseudoinsulata* Rego Barros (Lepidoptera: Erebidae) [12]. However, due to the poor distribution [13] and limited impacts of *P*. *pseudoinsulata* on *C*. *odorata*, calls for the introduction of additional effective herbivores were made [6]. In 2003, another biological control agent, the stem-galling fly, *Cecidochares connexa* (Macquart) (Diptera: Tephritidae), was successfully introduced into Côte d’Ivoire (R. Desmier de Chenon, personal communication to C. Zachariades) following its successful introduction and control of the weed in parts of Asia and Oceania [6]. The gall fly was originally collected from Colombia and was tested for host specificity in Indonesia prior to its release there [14].

Following egg-laying into the terminal and axillary buds of *C*. *odorata* stems, larval feeding induces gall formation [14]. The development of galls on *C*. *odorata* stems is detrimental to the vegetative and reproductive capacity of the plant because of the translocation of nutrients away from growth, flowering, and seeding [14, 15]. Consequently, the plant suffers dieback due to the deprivation of its nutrients [14, 15].

In 2014, *C*. *connexa* was reported in Ghana [16], and in Liberia and Guinea in 2016 [17], despite not ever having been released in these three countries. By 2016, it was reported throughout southern Ghana [18], albeit with some level of parasitism [19]. Nevertheless, the gall fly was having a significant negative influence on the vegetative growth of *C*. *odorata*, with a corresponding negative effect on the reproductive performance [20].

The gall fly was subsequently reported in Togo in 2016 [18] and Benin Republic in 2018 (P. Neuenschwander pers. comm. 2018). However, the gall fly was not seen in Nigeria at this time and its distribution in Benin Republic was poorly understood. Because biological control agents, as with invasive plants, can spread once introduced into novel environments, the need for surveys in Nigeria and Central Africa to determine whether *C*. *connexa* was present there as well has been documented [18]. Documenting the spread of biological control agents is important as it negates the need for countries wishing to undertake biological control to import effective biological control agents if they are already present. This paper reports for the first time the presence of *C*. *connexa* in Cameroon, as well as in new locations in Benin Republic, Ghana, Nigeria, and Togo, where the gall fly had not been previously reported. The implications of the significant expansions in the range of *C*. *connexa* across and beyond West Africa are discussed.

## Materials and methods

### Distribution of *Cecidochares connexa* in West Africa and Cameroon

**Fig 1:**
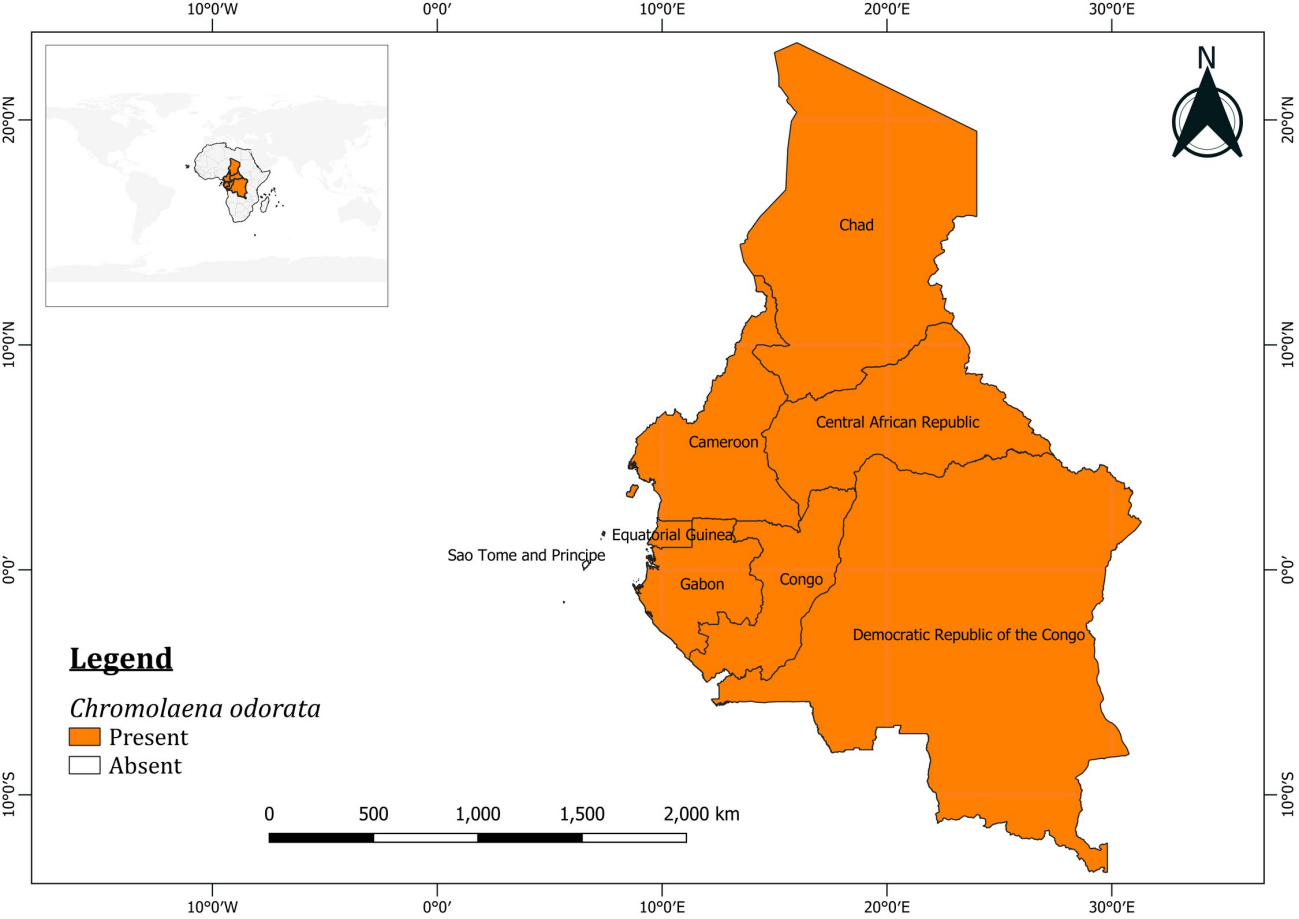
Occurrence and distribution of *Chromolaena odorata* in Central Africa. Only São Tomé and Príncipe has no record of the plant in the region. This figure was generated in QGIS (version 3.16.1 Hannover) https://qgis.org/downloads/ and the source files were obtained from Natural Earth https://www.naturalearthdata.com/.

*Chromolaena odorata* occurring in both fragmented patches and clutched densities were surveyed across 35 study locations in West Africa and Cameroon for the presence of *C*. *connexa* between October 2018 to October 2020. See S1 Table for further details. Although *C*. *odorata* occurs in all Central African countries except São Tomé and Príncipe [21–23] (Fig 1), the surveying of *C*. *connexa* in the region was restricted to Cameroon because the country borders Nigeria and is considered the potential entry point into Central Africa. *Chromolaena odorata* in six locations (Duaola, Kumba, Mamfe, Manyu, Mbengwi, and Yaoundé) in Cameroon were surveyed for *C*. *connexa* galls. These locations were selected based on preliminary surveys, conducted in Cameroon in 2018, showing the occurrence of *C*. *odorata* in these locations.

In addition to the surveys in Cameroon, *C*. *odorata* was surveyed for *C*. *connexa* galls in one location in Ghana and four in Nigeria, where no galls were recorded in surveys conducted in 2016. As surveys conducted in Togo in 2016 showed the presence of galls in two locations, Badja and Noepe [18], we purposively selected these two sites, in addition to seven new locations to confirm the status and distribution of the gall fly in the country. In Benin Republic, surveys of *C connexa* were conducted in 15 locations where no prior surveys had been undertaken.

Vegetation types surveyed encompassed *C*. *odorata*, grasses, trees, and shrubs. In the majority of the locations, *C*. *odorata* was surveyed along roadsides because these sites were easily accessible and proximal to the nearest village or town. In other cases, farmlands, abandoned plots, and residences that had varying densities of *C*. *odorata* were surveyed for *C*. *connexa*.

The presence of *C*. *connexa* galls was determined by visual examination of *C*. *odorata* stems at each location following permits by landowners, farmers, and head of households or institutions in each instance. Plants in an area of about 100m^2^ were sampled by a team of three persons for approximately four hours per location.

### Abundance of *Cecidochares connexa* in sampled areas in Cameroon

*Cecidochares connexa* was observed at five of the six locations in Cameroon where *C*. *odorata* was reported during the surveys. The name of each location where the gall fly was found was transcribed to a small ballot paper for random selection of three sites (Manyu, Mbengwi, and Kumba) to investigate the abundance of the gall fly over time. A stratified sampling method by Farrell and Lonsdale [24], albeit modified by Aigbedion-Atalor et al. [18], was used to estimate the abundance of *C*. *connexa*. Five 1m^2^ quadrats, made from polymerizing vinyl chloride, each spaced 20 m apart, were laid within a 100m line transect area in each of three locations. Sampling was conducted monthly from February to September 2019, and again from May to October 2020. In each quadrat, the number of *C*. *connexa* galls present was recorded. However, to prevent an overestimation of the population of *C*. *connexa* or recounting galls, only young galls, or galls with epidermal sealed emergence ‘windows’, indicating the non-occurrence of adult eclosion, were counted during each sampling event. This method preserves the integrity of accurately recording the growth of the population of *C*. *connexa* at any given time [18].

The same sampling methods described above were previously used in estimating gall densities in three locations each in the Eastern region of Ghana (Aburi, Somanya, and Kpong) and southwestern Togo (Badja, Kovie, and Noepe) in studies conducted in 2015 and 2016. Gall densities in Cameroon were then compared to the West African data to determine if similarities in gall densities occurred and for extrapolations on future gall densities.

### Statistical analysis

All count data (i.e. total number of galls per location) were fitted on quantile-quantile (q-q) plots. Shapiro’s test for normality and Levene’s test for variance homogeneity were conducted. As data did not follow the assumptions of normal distribution models (Shapiro-Wilk: *P* < 0.05), and variances were not homogenous (Levene’s test: *P <* 0.05), a generalized linear model (GLM) with a negative binomial distribution and log link function was used to test the interaction(s) (i.e. location x year x gall density) and differences in the number of galls recorded per location and over time in Cameroon. The significance of the model was determined by an analysis of deviance (with chi-squared test), while the Akaike information criterion (AIC) was used as an estimator to test the relative quality of the model. Both the analysis of deviance and AIC were used as validation in rejecting two previously tested GLM models (Poisson regression and Quasi-likelihood) that were deemed inappropriate. In instances where significant differences were detected, Tukey’s HSD test was used for mean separation. Since data on the change in the population density of the gall fly over time (i.e. from 2019 and 2020) were not normally distributed, a non-parametric paired Mann-Wilcoxon rank-sum test was performed. Variations in gall densities between the three locations each in Cameroon, Ghana, and Togo did not violate the assumptions of parametric tests (Shapiro-Wilk test and Levene’s test: *P >* 0.05). Gall densities recorded in each country (i.e. densities in 2015 and 2016 in Ghana, only 2016 in Togo, and densities in 2019 and 2020 in Cameroon) were pooled and then subjected to a one-way analysis of variance (ANOVA) to test for significant differences in gall abundance in the three countries. Following the detection of a significant test, Student-Neuman-Keuls (SNK) *post hoc* test was performed for mean separation. All statistical analyses were performed in the R (version 3.5.1) environment for statistical computing [25].

## Results

### Distribution of *Cecidochares connexa* in West Africa and Cameroon

**Fig 2:**
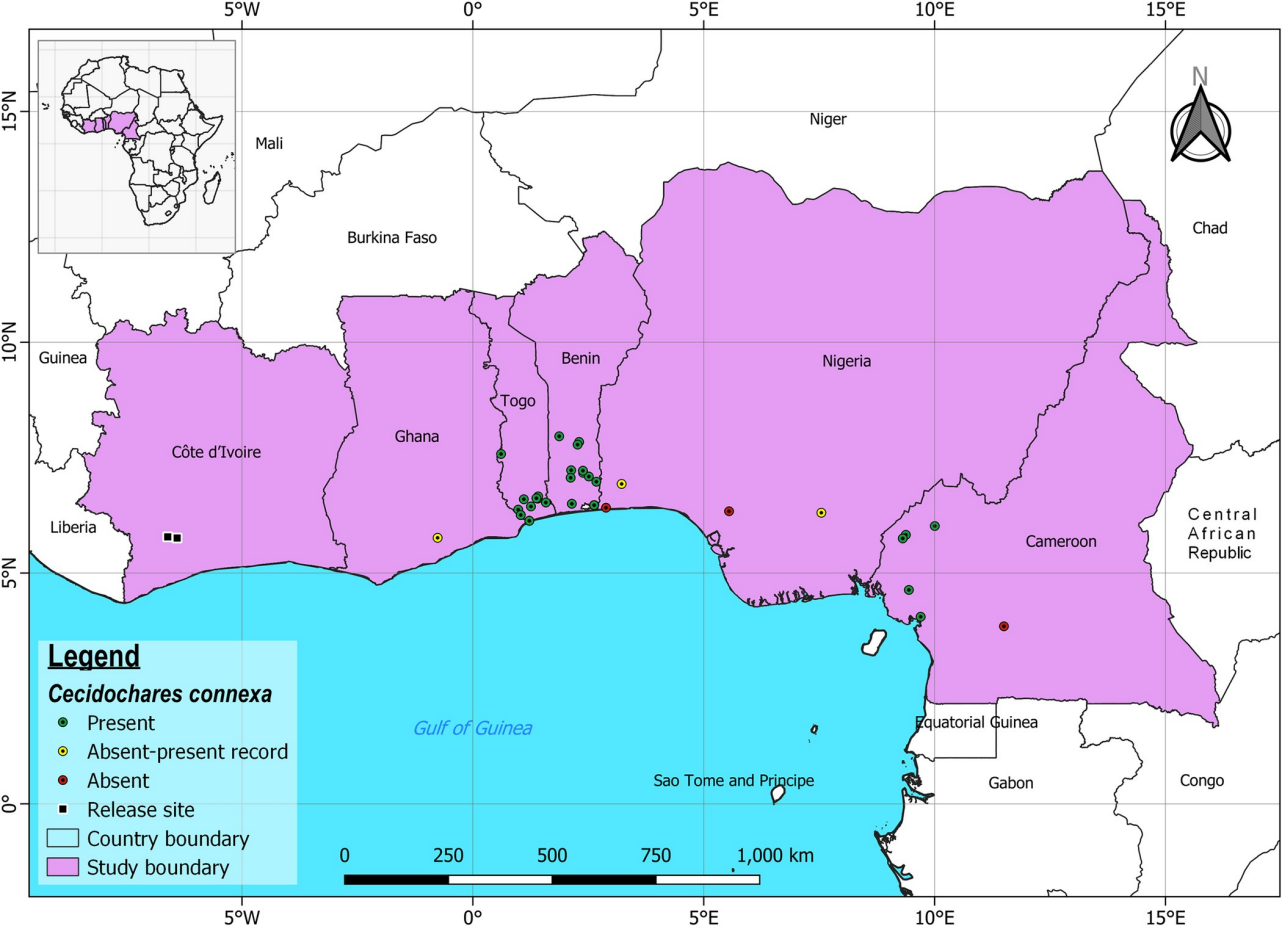
Occurrence of *C. connexa* in Ghana, Togo, Benin and Nigeria (West Africa) and Cameroon (Central Africa) in the sampling efforts from 2018 to 2020. Absent-present records indicate locations where galls were not seen in 2016 but were observed in the current surveys. See (S1 Table) for further details. This figure was generated in QGIS (version 3.16.1 Hannover) https://qgis.org/downloads/ and the source files were obtained from Natural Earth https://www.naturalearthdata.com/.

Galls of *C*. *connexa* were recorded in five of the six locations surveyed in Cameroon, Central Africa. No galls were recorded in Yaoundé (Fig 2). In Benin Republic, although densities of the gall fly were not assessed, *C*. *connexa* galls were widespread and easy to detect in all 15 locations sampled, albeit with apparent varying levels of abundance. In Nigeria, *C*. *connexa* galls were recorded in two locations: Ewekoro (6°55’56.8’’N, 3°13’09.3’’E) in Ogun State and Agbani (6°18’29.9’’N, 7°32’52.8’’E) in Enugu State, out of the four locations where no galls were seen when previously surveyed in 2016 (Fig 2). No galls were found in Evbuotubu (6°20’25.6’’N, 5°32’56.1’’E) in Edo State or Badagry (6°25’01.1’’N, 2°53’00.3’’E) in Lagos State. In Ghana, galls were recorded in Akroso (5°45’54.6’’N, 0°45’46.2’’W) in the Eastern region of Ghana, where they were not previously recorded in 2016. Galls were also recorded in Noepe (6°15’37.7”N, 1°02’13.5”E) and Badja (6°22’34.3”N, 0°59’08.4”E), in Togo where *C*. *connexa* was reported present in 2016, as well as seven new sites not previously surveyed (Fig 2).

### Abundance of *Cecidochares connexa* in the sampled areas in Cameroon

**Fig 3:**
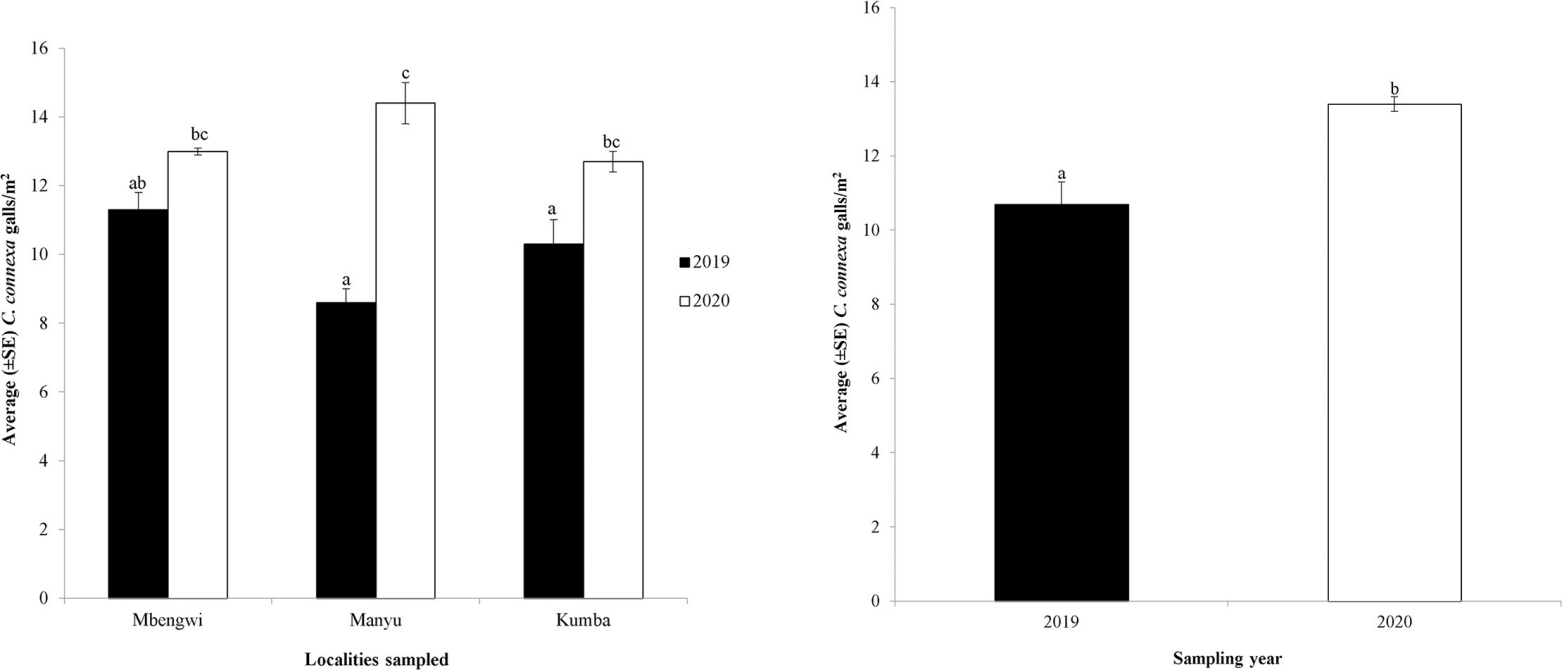
(A) Establishment and mean population density (±SE) of *C*. *connexa* galls in the three locations (Mbengwi, Manyu and Kumba) sampled in Cameroon in 2019 and 2020 and (B) cumulative change in the population density of *C*. *connexa* in Cameroon between 2019 and 2020. Error bars with lowercase letters compared yearly variations in gall density within and between locations, respectively. Error bars that do not have at least one identical lowercase letter indicate significant difference following a generalized linear model (GLM) and Tukey’s HSD, *P* < 0.05.

*Cecidochares connexa* galls were observed in Manyu, Mbengwi, and Kumba in both 2019 and 2020. There was a significant interaction between gall density, locations in Cameroon, and time (i.e. year) (GLM, *χ*^2^ = 72.01, df = 2, P = 0.005) (Fig 3A). Mbengwi had the highest density of galls in 2019 ($\bar{x}$ = 11.3), albeit not significantly different from Kumba ($\bar{x}$ = 10.3) or Manyu ($\bar{x}$ = 8.6) (Fig 3A). In 2020, gall density was highest in Manyu ($\bar{x}$ = 14.4) but did not differ significantly from that in the other two locations (Fig 3A). Cumulatively, *C*. *connexa* gall densities were significantly higher in 2020 than in 2019 (*U* = 167, *P* = 0.013) (Fig 3B). Within each location, only Mbengwi did not have *C*. *connexa* gall densities that were significantly higher in 2020 than in 2019 (Fig 3A).

**Fig 4:**
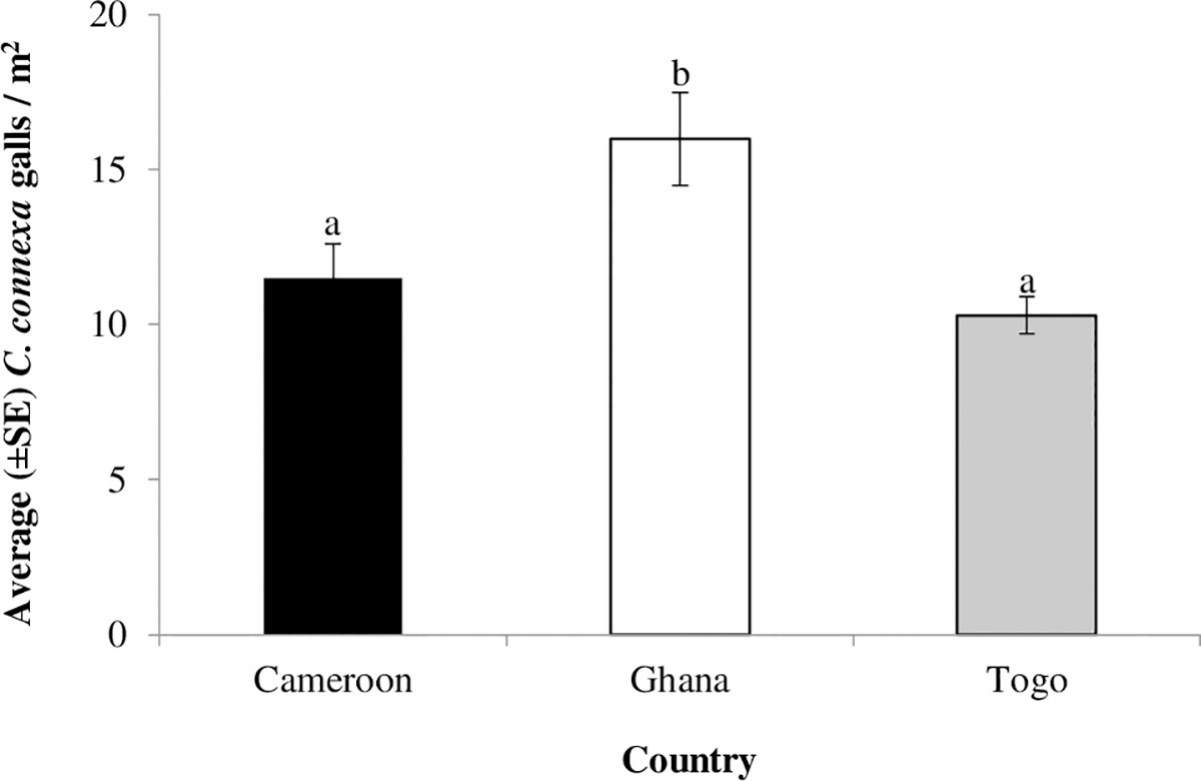
Density (±SE) of *Cecidochares connexa* in the sampled areas of Cameroon, Ghana, and Togo. Locations (Aburi, Somanya, and Kpong) sampled in Ghana in 2015 and 2016 are from the Eastern region, while locations (Badja, Kovie, and Noepe) sampled in Togo in 2016 only are from the southwestern part of the country. Manyu, Mbengwi, and Kumba were sampled in Cameroon in 2019 and 2020, respectively. Error bars with lowercase letters compared variations in gall density between countries and different letters indicate significant difference following a one-way ANOVA and Student-Neuman-Keuls (SNK) test, *P* < 0.05.

Regionally, gall density ($\bar{x}$ = 11.5) in Cameroon was significantly different from densities in Ghana ($\bar{x}$ = 16.1) but similar to densities in Togo ($\bar{x}$ = 10.3) (F_2,35_ = 4.75, *P* = 0.020). Overall, Ghana had more galls than Cameroon and Togo (Fig 4).

**Fig 5:**
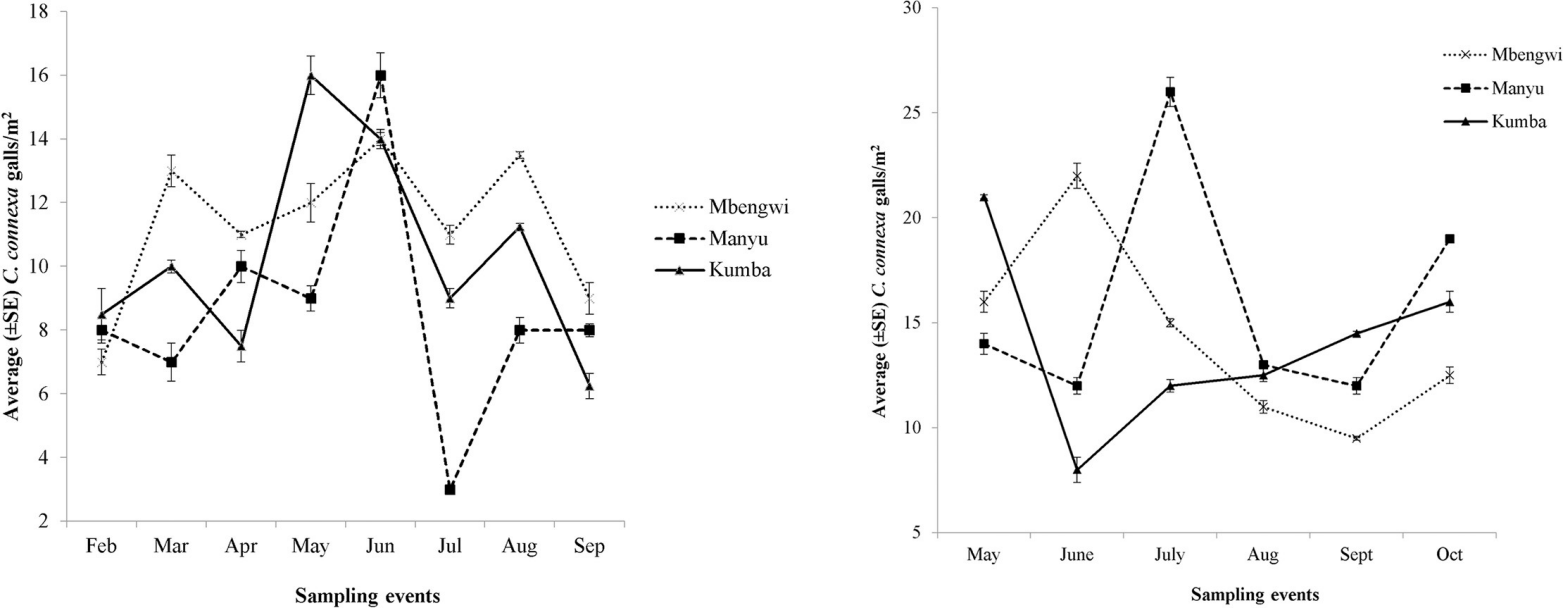
Spatiotemporal distribution of *Cecidochares connexa* in Manyu, Mbengwi, and Kumba in Cameroon (A) from February to September 2019 and (B) May to October 2020. The same sampling technique and gall sampling parameters were adopted in each location and across time.

The spatiotemporal distribution of galls in each of the three study locations in Cameroon had different density oscillatory trends (Fig 5A and 5B). In 2019, galls peaked in Manyu, Mbengwi and Kumba in June, March, and May, respectively (Fig 5A), while gall trends in 2020 peaked in Manyu, Mbengwi and Kumba, generally later, in July, June, and May, respectively (Fig 5B).

## Discussion

The spread of *C*. *connexa* from West Africa into Central Africa is documented for the first time and represents the first report of the gall fly outside of West Africa within the African continent. Also, there is evidence showing significant expansion in the known distribution range of *C*. *connexa* in West Africa, as reflected in the new reports of its distribution in Togo, Benin Republic, Ghana, and Nigeria. Overall, it has taken five years (2015 to 2019 inclusive) of extensive post-release surveys since *C*. *connexa* was first recorded outside of Côte d’Ivoire by Paterson and Akpabey [16], to observe and document its dispersal across West Africa and into Cameroon in Central Africa. Currently, *C*. *connexa* is confirmed present in seven West African countries (Benin Republic, Côte d’Ivoire, Ghana, Guinea, Liberia, Nigeria, and Togo) and one Central African country (Cameroon). The estimated distance the gall fly has moved following its release in Côte d’Ivoire in 2003 is over 2,300 km.

Although we are not sure of the exact time of the spread of the gall fly into Cameroon, it may have been present in the country for some time, given the numbers of galls seen in the five locations, but just not detected by anyone. Gall numbers were similar to those recorded in Togo, where it was first detected in 2016. However, numbers were significantly lower than those in Ghana where the gall fly was first detected in 2014. Also, the change in the density of the gall fly in Cameroon between 2019 and 2020, confirming its establishment, is somewhat similar to the change in gall densities documented in Ghana after the first two years (2015 and 2016) of its report in that country [16, 18]. Furthermore, the increase in gall density in the sampled areas of Cameroon over time is consistent with the reports that *C*. *connexa* readily establishes once it disperses into climatically suitable environments. These findings suggest that the gall fly may have dispersed into Cameroon before 2019 and may be present in other countries in Central Africa.

The distance spread by *C*. *connexa* is not unusual for biological control agents. *Ophiomyia camarae* Spencer (Diptera: Agromyzidae), an agent released against *Lantana camara* L. (Verbenaceae), has now spread to 11 countries, including Madagascar and Ethiopia, following its release in South Africa in 2001 [17]. Having an innate ability for long-distance dispersal is crucial to the success of all biological control agents [26, 27]. This is because when agents spread within and far away from areas of their release, they often do so by significantly increasing their population which, in most cases, increases the number of genetically fit (larger in size and more fecund) individuals in the population. In almost all cases, this phenomenon improves the overall impacts of biological control agents, as they can track the range expansion of their target hosts [28]. Evidently, this is the case with *C*. *connexa* in West Africa, as well as in many areas in Southeast Asia and Oceania, where it has been or is currently being successful in controlling *C*. *odorata* [5, 17, 18, 20, 28–30].

No doubt, the host-seeking behaviour of *C*. *connexa* also contributes significantly to its success against *C*. *odorata*, as it has the ability of searching and ovipositing into *C*. *odorata* stems in landscapes where the plant occurs in clutched densities or fragmented patches. Put simply, its ability to find *C*. *odorata* in landscapes where the plant occurs in fragmented patches is fundamental for its long dispersal. It is now well-articulated that even when landscapes with highly dense *C*. *odorata* are separated by long gaps of little or fragmented *C*. *odorata* patches, *C*. *connexa* could still build up densities in those areas on the few available plants and then disperse elsewhere.

The impacts of *C*. *odorata* in Central Africa are similar to those in West Africa [9, 31]. There is evidence that *C*. *odorata* is detrimental to the establishment of native plant species in fallow lands in Cameroon [31]. Tchiengue et al. [31] reported that the persistent occurrence of *C*. *odorata* thickets in vegetation prevents succession by native species by creating a blockade that limits the deposition of external propagules of native plant species. The latter authors alluded that in the forest regions of Cameroon, *C*. *odorata* induces significant delays in forest recovery and succession and acts as the key factor that explains low species richness and diversity, thus following the inhibition model of Connell and Slatyer [32]. This suggests that the formation of a highly intricate matrix that prevents further colonization by native species is due to the high levels of the initial abundance of *C*. *odorata* in Cameroon either in the early 1930s [6, 33] or in the 1960s [6, 34]. In fact, this is the scenario in the entire invaded range of *C*. *odorata*, which makes control efforts a complex matter.

In other parts of Central Africa, such as the Democratic Republic of the Congo, dense infestations of *C*. *odorata*, producing myriad seeds, have been reported [6]. Since the spread of *C*. *odorata* in the Central African Republic in the 1980s, the plant has been a serious threat to native species, especially in cleared forest ecosystems [33]. Given that current management options for *C*. *odorata* are costly, time-consuming, and not sustainable, the spread of *C*. *connexa* into Cameroon is crucial, timely and opportunistic for the management of *C*. *odorata*.

In studies conducted elsewhere [14, 15, 28, 29], *C*. *connexa* has the ability to reduce growth and flowering of *C*. *odorata*, and ultimately reduce densities of *C*. *odorata* by 90%, and offers long-term suppression of the plant without augmentative releases [5, 28, 29]. For example, following the introduction of *C*. *connexa* into Papua New Guinea in 2001, it readily established in all provinces in which it was released then dispersed to new areas, covering all areas where *C*. *odorata* occurs within those provinces [29]. By 2013, it had reduced the cover of the plant by over 90% [28]. Similarly, the gall fly has significantly reduced *C*. *odorata* cover and impacts in Timor Leste [30], Indonesia, and India [17]. In West Africa, the gall fly is causing significant dieback of *C*. *odorata* and reductions in seed production [20].

It is anticipated that *C*. *connexa*, once established, will have similar impacts to *C*. *odorata* in Central Africa. Given the current climatic suitability of the gall fly in Central Africa (Aigbedion-Atalor et al., unpublished data), we anticipate that the gall fly will continue to naturally disperse into new areas in the region, providing relief for farmers and land managers, as well as reducing impacts of *C*. *odorata* on biodiversity. However, considering that *C*. *connexa* is a relatively recent agent in Central Africa, a considerable amount of time–up to ten years–may be required for it to cause significant reductions in the overall cover of *C*. *odorata* in the region. Therefore, to expedite the impacts of the gall fly on *C*. *odorata* in Central Africa, a regional concerted effort focusing on the redistribution of galls for the biological control of *C*. *odorata* could be considered. In this regional biological control programme against *C odorata*, surveying for *C*. *connexa* could be conducted countrywide in Cameroon and across Central Africa to determine its current distribution and abundance in the region. These efforts could also underscore the cover of *C*. *odorata* and rank impacts thereof. *Cecidochares connexa* galls could be collected from Cameroon and in other potential areas where the gall fly occurs in the region, mass-reared, and then introduced into other parts of the region, especially in areas where the density cover and impacts of *C*. *odorata* are unabated. These areas may include ecosystems such as farmlands, protected areas, forest margins, and botanical gardens. Alternatively, galls from sites where *C*. *connexa* is well-established could be collected and introduced directly into other areas as it is a less expensive, efficient, and easier re-distribution method than mass-rearing galls in laboratories. This has been proven in Papua New Guinea where as few as 100 galls, collected from sites where the gall fly occurs, were used to establish *C*. *connexa* in new areas [28, 29]. Yearly post-release monitoring and evaluation of the gall fly could then be considered. These data (i.e. post-release evaluation) when compared with pre-release data on *C*. *odorata* cover and impacts may show empirical evidence of the impacts of the gall fly.

## Supporting information

S1 Table Study countries, geographic information, and status of *Cecidochares connexa*. (DOCX)
