# Supplementary material for: Marching across and beyond West Africa: First record of the stem-galling fly Cecidochares connexa (Diptera: Tephritidae) in Central Africa and the implications for biological control of Chromolaena odorata (Asteraceae)
Source: PLoS One. 2021 Jun 4;16(6):e0252770. doi: 10.1371/journal.pone.0252770 (PMC8177621; doi:10.1371/journal.pone.0252770)
Supplement: S1 Table — (DOCX) [file pone.0252770.s001.docx]

| **S1 Table. Study countries, geographic information, and status of *Cecidochares connexa*** | | | |
| --- | --- | --- | --- |
| **Country** | **Longitude** | **Latitude** | ***C. connexa*** |
| Benin | 1.87018 | 7.96413 | Present |
| Benin | 2.62652 | 6.47216 | Present |
| Benin | 2.30208 | 7.8419 | Present |
| Benin | 2.38882 | 7.1736 | Present |
| Benin | 2.67535 | 6.98025 | Present |
| Benin | 2.62652 | 6.47216 | Present |
| Benin | 2.62652 | 6.47216 | Present |
| Benin | 2.62652 | 6.47216 | Present |
| Benin | 2.26849 | 7.78388 | Present |
| Benin | 2.14325 | 6.50262 | Present |
| Benin | 2.12918 | 7.22841 | Present |
| Benin | 2.51122 | 7.09346 | Present |
| Benin | 2.38433 | 7.21912 | Present |
| Benin | 2.11867 | 7.06698 | Present |
| Benin | 2.23905 | 7.96926 | Present |
| Ghana | -0.76284 | 5.76518 | Present |
| Cameroon | 9.37738 | 5.82876 | Present |
| Cameroon | 9.44347 | 4.63602 | Present |
| Cameroon | 9.69872 | 4.05486 | Present |
| Cameroon | 10.00477 | 6.02027 | Present |
| Cameroon | 9.31094 | 5.75194 | Present |
| Nigeria | 3.22204 | 6.93244 | Present |
| Nigeria | 7.54798 | 6.30831 | Present |
| Togo | 1.03685 | 6.2591 | Present |
| Togo | 0.98302 | 6.37606 | Present |
| Togo | 1.03685 | 6.2591 | Present |
| Togo | 1.22311 | 6.13365 | Present |
| Togo | 1.22311 | 6.13365 | Present |
| Togo | 1.10422 | 6.60047 | Present |
| Togo | 0.61097 | 7.58166 | Present |
| Togo | 1.40309 | 6.61306 | Present |
| Togo | 1.4144 | 6.6627 | Present |
| Cameroon | 11.50208 | 3.848032 | Absent |
| Nigeria | 5.54893 | 6.34043 | Absent |
| Nigeria | 2.88343 | 6.41697 | Absent |
